# Is synaesthesia a dominantly female trait?

**DOI:** 10.1080/17588928.2015.1019441

**Published:** 2015-03-09

**Authors:** Julia Simner, Duncan A. Carmichael

**Affiliations:** 1 School of Psychology, Pevensey Building, University of Sussex, Falmer, UK; 2 Department of Psychology, University of Edinburgh, Edinburgh, UK; 3 Institute for Adaptive & Neural Computation, University of Edinburgh, Edinburgh, UK; 4 Division of Psychiatry, University of Edinburgh, Royal Edinburgh Hospital, Edinburgh, UK

**Keywords:** Synaesthesia, Prevalence, Sex ratio, Synesthesia

## Abstract

*Synaesthesia* is a familial condition that gives rise to unusual secondary percepts. We present a large-scale prevalence study which informs our ideas on whether the condition is more prevalent in men or women. A number of studies over the last 20 years have suggested the condition is found more commonly in women, with up to six times more female synaesthetes than male. Other studies attributed this female bias to merely a recruitment confound: women synaesthetes may be more likely to self-refer for study. We offer two pieces of evidence that there is no extreme female bias in synaesthesia: first we re-analyse previous reports of very large female biases to show again that they likely arose from self-referral or other methodological issues. Second, we present the largest published prevalence study to date on *grapheme→colour synaesthesia* in which our prevalence (1.39% of the population) replicates our earlier estimates (and in which we demonstrate no strong female bias even with sufficient power to detect such a difference.

For people with synaesthesia, stimuli are experienced with unusual secondary associations (e.g., hearing sound triggers colours in the visual field; Ward, Huckstep & Tsakanikos, 2006). Synaesthesia is a multi-variant condition with an estimated 65 (Day, 2005) to 150 (Cytowic & Eagleman, 2009) known sub-types, depending on which modalities are linked (e.g., sound triggering colours, taste triggering touch etc.). One key question is how common synaesthesia is, and whether it affects men and women differently. Early estimates described the condition as extremely rare (e.g., 1 in 250,000) and very strongly female dominant (with a 6:1 ratio; Baron-Cohen, Burt, Smith-Laittan, Harrison & Bolton, 1996). Later studies have called into question both these claims and we examine these issues in the current paper.

Despite a relatively contentious history, the question of synaesthesia’s prevalence appears to now be reasonably well understood. Early estimates of prevalence varied widely at least partly because researchers were focussing on different sub-types or using different definitional criteria (Ramachandran & Hubbard, 2001). However, even in studies that aimed to report the prevalence of all forms of synaesthesia, estimates ranged from 1 in 4 (Calkins, 1895; Domino, 1989; Uhlich, 1957), to 1 in 10 (Rose, 1909), 1 in 20 (Galton, 1883), 1 in 200 (Ramachandran & Hubbard, 2001), 1 in 2000 (Baron-Cohen et al., 1996), and 1 in 25,000–100,000 (Cytowic, 1993, 1997). One problem was that many of these early estimates were essentially ‘best guesses’. Nonetheless, these early studies served the important purpose of stimulating research on synaesthesia’s prevalence and inspired the first empirical assessments which followed thereafter.

The first prevalence study of its kind in the modern literature (Baron-Cohen et al., 1996) assessed the occurrence of synaesthesia by placing adverts in two local newspapers in Cambridge, UK, calling for synaesthetes to come forward. The advert described several types of synaesthesia (sound [including ling-uistic sounds]→colour, touch/taste/smell→vision/sound) and identified two types of synaesthesia in respondents, now known commonly as *grapheme-colour synaesthesia* (experiencing colours from letters and/or digits) and *music-colour synaesthesia* (experiencing colours from sounds such as music). By comparing the number of synaesthetes who came forward (and who were subsequently verified as genuine using an objective test; see below) against the circulation figures of the newspapers, Baron-Cohen and colleagues concluded that synaesthesia was at least as common as 1 in 2000 people (i.e., 0.05%). However, their methods would have greatly underestimated the true prevalence because they relied on synaesthetes making the effort to come forward in response to a newspaper advert. For this reason the authors of that study were careful to point out that their figure was only a lower estimate, although their data has almost always been misrepresented in the following literature as an absolute estimate. A small number of studies in the historical literature had avoided the problems of self-referral by individually questioning every member of a participant pool, although they established prevalence only subjectively (at 6.7–23.0%: Calkins, 1895; Domino, 1989; Rose, 1909; Uhlich, 1957) by relying on self-declaration only, which is an approach known to over-estimate prevalence (Simner et al., 2006). Hence, one set of studies tends towards a conservative estimate and the others towards an overly-liberal one.

Our own study in 2006 addressed these limitations by individually assessing a large number of people (n = 1690^1^^1^Specifically, 1190 individuals were assessed for grapheme-colour synaesthesia, and a further 500 individuals were tested for 162 different synaesthesias, one also being grapheme-colour synaesthesia. Since the estimates of prevalence for grapheme-colour synaesthesia were approximately equivalent across both populations, Simner et al. (2006) collapsed both population sizes to give a grand total of 1690 people tested.) and verifying their self-declarations with an objective test of genuineness (see below). These improvements in methodology showed the condition to be far more common than previously thought, affecting 1 in 23 members of the general population across the relatively wide range of synaesthesias tested (Simner et al., 2006). The important element in this study was that synaesthetes were not required to make the effort to self-refer in response to an advert. Instead, a large sample of the general population was individually assessed to find the synaesthetes from among them, and this gave a prevalence of 4.4% of synaesthetes within the general population, for the variants tested. Within this figure, one particularly common form was grapheme-colour synaesthesia, in which colours are triggered by letters and/or digits (e.g., *A* might trigger the experience of red, *B* yellow, and so on). The prevalence of grapheme-colour synaesthesia was 2% (counting those with coloured letters *and/or* digits; or 1.1–1.4% for those with both coloured letters *and* digits). Since the time of this study, these estimates for the prevalence of different forms of synaesthesia have been widely accepted (e.g., Banissy et al., 2012; Bor, Rothen, Schwartzman, Clayton & Seth, 2014; Cohen Kadosh & Henik, 2007; Ward, 2013; Weiss & Fink, 2009).

In contrast to prevalence estimates, the sex ratio of female to male synaesthetes has caused perhaps greater controversy. Several early studies proposed that there was a very strong female bias in synaesthesia, suggesting a possible X-linked dominant mode of inheritance (e.g., Baron-Cohen et al., 1996; Smilek et al., 2002). Indeed, the extent of this female bias in some studies (e.g. 6:1; Baron-Cohen et al., 1996) led researchers to believe that the trait may even be associated with male lethality *in utero* (Bailey & Johnson, 1997; Baron-Cohen et al., 1996). This would in turn suggest that synaesthetes’ families should contain more women than men. However, both these claims were subsequently challenged by later studies, and we describe this development below.

Until 2006, the most commonly cited synaesthesia study on prevalence and sex-ratios (Baron-Cohen et al., 1996) proposed a female: male ratio of 5.5:1, and this was followed by a second study (Rich, Bradshaw & Mattingley, 2005) proposing a female bias of 6.2:1. However, both studies based their estimates on the number of synaesthete who self-referred in response to media advertisements (e.g., newspaper adverts). Not only will this method underestimate the total number of synaesthetes in a population (see above) but it is also likely to over-estimate the females. This is because females are known to be more likely than males to come forward to report atypical experiences, and this is seen across a range of domains (e.g., Dindia & Allen, 1992). Simner et al. (2006) therefore suggested that a self-referral confound may be responsible for the previously high rates of female synaesthetes in prevalence studies. Indeed, when this potential confound was directly avoided by Simner et al. (2006), we found that earlier studies had indeed apparently over-inflated the proportion of females. As noted above, Simner et al. (2006) specifically did *not* rely on self-referred recruitment in their prevalence estimate, but instead, they individually assessed every member of a large participant pool and used an objective test to identify the synaesthetes from among them. Using this improved methodology we found that that there was no large (e.g., 6:1) bias towards female synaesthetes. Instead, we found a female: male ratio of 1.1:1 when considering a wide range of synaesthesias in a population of n = 500, and a female: male ratio of 0.9:1 when considering grapheme-colour synaesthesia^2^^2^This particular reported figure related to grapheme-colour synaesthetes who experience both coloured letters *and* digits (rather than coloured letters and/or digits). in a population of 1190. Neither of these comparisons showed any significant sex bias.

On the basis of the above literature we might conclude that synaesthesia affects around 1 in 23 individuals and has no very strong sex bias. However, there have been three subsequent challenges to our position. First, a small number of studies continue to cite the prevalence and/or sex ratio from Baron-Cohen et al. (1996) despite the self-referral confound, and this has propagated in the literature a low value of prevalence and a high estimate of female synaesthetes. Second, one subsequent study (Barnett, Newell, Finucane, Asher, Corvin, Mitchell, 2008) has pointed out that the sex differences identified in self-referral more generally (Dindia & Allen, 1992) only account for a slight variation (10%) in men and women’s responding, making it possible that very high early estimates for female synaesthetes were at least pointing in the right direction. Third, that same study (Barnett et al., 2008) presented data that were ostensibly free from the self-referral confound, but which continued to show a strong (6:1) ratio of female to male synaesthetes. For these three reasons we return to the issue of sex differences in synaesthesia in the current paper. The position we take is to re-affirm that there is no strong 6:1 ratio of female to male synaesthetes when all self-referral confounds are removed. We do this below by presenting our own very large-scale study free of self-referral, but before then, we also re-evaluate the findings by Barnett et al. (2008). Their findings had been reported as evidence of a strong (6:1) ratio of female to male synaesthetes in data that were presented as being apparently free from the self-referral confounds. We re-evaluate this claim below.

Barnett and colleagues conducted a synaesthesia study of the mode of inheritance, and prevalence of synaesthesic sub-types within families. In their study they looked not only at self-referred probands (i.e., 53 synaesthetes who self-referred to the university in response to a media advert) but also a subset of their family members who were questioned by the proband and/or directly contacted by the researchers. Since family members were tested as well as self-referred probands, Barnett et al. claim their findings are free of a self-referral confound, and they report that “our total sample of 92 confirmed and unconfirmed synaesthetes includes 78 females and 14 males, yielding a female to male ratio of 6:1 in the Irish population” (pg. 877). Below we present several responses to these claims.

First, in their calculations of the female: male ratio, Barnett et al. appear to directly compare their 78 female synaesthetes against their 14 male synaesthetes, concluding that a female: male ratio in synaesthesia of 6:1 exists in the general population (more precisely this would be: ^78^/_14_: ^14^/_14_ = 5.57:1). However, Barnett et al. evaluated twice as many females than males (118 vs. 61) if we include all 179 participants whose status was somehow appraised during their study (i.e., excluding all those with an unknown status). This factor would considerably reduce the absolute proportion of female synaesthetes to males in their estimate for the general population.

A second consideration comes in the claim that Barnett et al.’s findings were not contaminated by a self-referral bias, because they looked not only at self-referred probands but also their families. However, according to our reading of their report, Barnett and colleagues were able to objectively verify the synaesthesia of all their self-referred probands, but only a small portion of their non-proband synaesthetes. Indeed, 81 of their 92 synesthetes overall were either objectively unconfirmed cases (n = 28), or they self-referred in response to an advert (n = 53) and were therefore likely to be *a priori* female-skewed. Equally, when Barnett et al. state there was “no difference… between the sex ratio for probands (46 females and 7 males) and … relatives who did not contact us directly (30 females and 5 males)”, we point out that almost 70% of these synaesthete relatives appear to have received no objective test for synaesthesia. As such, almost every member of their cohort were either self-referred, or were not verified as synaesthetes by the usual objective standard.

Finally, Barnett et al. (2008) report that 17 families were fully explored as far as all first-degree relatives of the proband and this still gave a “6:1” (pg 885) ratio of female to male synaesthetes (i.e., 45 female synaesthetes and 8 male synaesthetes found within these 17 families). We point out, as above, that 45 female vs. 8 male synaesthetes cannot be interpreted as 6:1 prevalence in the general population without first knowing the sex of each family member tested in those 17 families as a whole which was not provided. We also point out that one third of the synaesthetes discovered within those 17 families (i.e., 17 of the 53 synaesthetes discovered) would have been contaminated by a self-referral confound that strongly skews towards females, because these families centred around 17 synaesthete probands, who self-referred for study. Importantly, 87% of all (n = 53) probands were female, meaning that approximately 87% of the 17 probands in the target families would be females, from what we know is a skewed sampling method. In summary, target families were not selected in a way to be free of an *a priori* recruitment confound because all contained a proband recruited by self-referral (see also Ward & Simner, 2005 for a similar problem). Finally, we point out, as above, that approximately half of the 53 synaesthetes within the 17 target families did not receive an objective test (of consistency) for synaesthesia.

In summary, we conclude that the 6:1 ratio towards female synaesthetes found by Barnett et al. (2008) did not take into account the total number of females categorised overall, or *a priori* confounds in the recruitment of self-referred synaesthetes, and it did not categorise synaesthetes with objective testing throughout. For these reason we conclude that their 6:1 bias towards female synaesthetes was affected, at least to some degree, by self-referral methodology or *other* issues. (Nonetheless, we point out that the study by Barnett and colleagues provided much robust data on *a number of other* epidemiological and cognitive factors within synaesthesia—e.g., transmission of different variants within families, trends in synaesthetic colours. etc.—and it therefore represents a valuable step towards understanding how synaesthesia might manifest itself, beyond this sex issue.) Below we test whether there is a 6:1 female bias empirically when self-referral is removed, but we first conduct a power analysis to confirm the numbers that would need to be tested in order to determine whether such a difference were statistically significant. This is important because previous epidemiological studies of synaesthesia aiming to remove the self-referral confound (e.g., Simner, Harrold, Creed, Monro & Foulkes, 2009; Simner et al., 2006) have tested too few people to provide sufficient power for a statistical comparison of the sexes.

## POWER ANALYSIS

The female bias in synaesthesia estimated by Baron-Cohen et al. (1996) was 5.5:1, and by Rich et al. (2005) it was 6.2:1, and by Barnett et al. (2008) it was 5.6:1. These values, repeatedly circling around a 6:1 ratio of female to male synaesthetes, can be tested empirically if there is sufficient power in the number of individuals tested. In order to calculate this we first need to estimate what the individual prevalences of synaesthesia would be for males versus females, given a hypothesised 6:1 difference.

The most robust and widely cited synaesthesia prevalence study to date (Simner et al., 2006), report an overall prevalence of synaesthesia of 4.4% of the population, when testing for 162 different variants. However, there are considerable challenges to identifying so many different types of sub-variants within a single study (see Simner et al., 2006 for discussion) so we instead chose to test for just one variant of synaesthesia in the current study. We chose grapheme-colour synaesthesia since this variant is very well understood, relatively prevalent, and can be tested for using a single standardised computerised method (see below). Below we therefore conduct a power analysis to reveal the number of individuals required for screening in order to identify any 6:1 bias of female synaesthetes with grapheme-colour synaesthesia.

Simner et al. (2006) report the prevalence of grapheme-colour synaesthesia to be 2% (where “grapheme-colour synaesthetes” are those with either coloured letters, coloured digits, or with both). With an assumed sex ratio of 6 female synaesthetes to every male synaesthete, we would expect to find 1.71 female synaesthetes and 0.29 male synaesthetes if we tested 100 members of the population. If we carry out a sample size calculation for a chi-squared test, with standard levels of power at 0.80 and alpha at 0.05, in order to detect a difference in proportion of this magnitude (1.71% versus 0.29%, or proportions of 0.0171 and 0.0029 respectively) a sample of 1810 participants is required for screening (905 females and 905 males). In our empirical study below, we meet—and indeed exceed—this sample size.

## EMPIRICAL ASSESSMENT

We individually assessed a very large number of individuals from the general population for grapheme-colour synaesthesia, avoiding a self-referral bias. Every person was assessed using the behavioural “gold-standard” test which identifies synaesthetes by detecting the most widely accepted core characteristic of synaesthesia. This characteristic is the consistency in the reporting of synaesthetic sensations over time. In grapheme-colour synaesthesia for example, a given letter tends to elicit a consistent synaesthetic colour for any given synaesthete in repeated testing (e.g., *A* might be consistently red, *B* consistently blue, etc.). This consistency-over-time is taken as the behavioural hallmark of synaesthesia in standard diagnostic tests for synaesthesia (see Johnson, Allison & Baron-Cohen, 2013 for review). The mostly widely used version of this test for grapheme-colour synaesthesia is available at an online interface known as the *Synesthesia Battery* (Eagleman, Kagan, Nelson, Sagaram & Sarma, 2007). In this test, participants are required to repeatedly report their synaesthetic associations for the letters A-Z and/or the digits 0–9, each shown three times in a random order. In order for people to be diagnosed as synaesthetes, they must achieve high enough consistency in their colour-choices to show they are significantly better than non-synaesthete controls, who previously performed the same test to provide a robust base-line. This task was used in our own study, and more details are given in Eagleman et al. (2007) and in our methods below.

### Participants

We individually screened 3893 participants for grapheme-colour synaesthesia using *The Synesthesia Battery* (2135 female; 1758 male). Their mean age was 28.3 years (SD = 14.2). A further 65 participants were excluded from study because they had entered an obviously false date of birth (e.g., 2013; n = 48) or because they reported too few coloured graphemes for their synaesthesia to be meaningfully evaluated (n = 17; see Eagleman et al., 2007). Participants were unpaid, and our study was approved by the local university ethics board.

Participants were recruited as part of a large-scale, centrally co-ordinated undergraduate research project, described in detail in Carmichael, Down, Shillcock, Eagleman and Simner (2015). In this, every student registered on the 2nd year of the Psychology undergraduate course at the University of Edinburgh between September 2012 and May 2015 acted as a research assistant (RA), each recruiting approximately 8 participants (4 male and 4 female) over 16 years of age. In recruiting participants, we took a number of steps to ensure as random a sample as possible: RAs were required to pre-select their sample, and then approach participants in a targeted way, rather than sending out an advert for self-referrals. Indeed, RAs were required to refrained from recruiting participants via any advert or open calls at all. For example, they could not post the testing URL on social media websites or internet forums. Furthermore, RAs were instructed not to deliberately seek out, nor to avoid, people they knew to be synesthetes and were also instructed not to *a priori* inform participants that the study investigated synesthesia. Instead, they pre-selected their samples to create a pre-determined, non-referred testing cohort, and then individually tested every member of that cohort.

### Methods

To screen for grapheme-colour synaesthesia, we used the consistency test from the *Synesthesia Battery* on-line interface (Eagleman et al., 2007), which we cloned with permission from the authors (see Carmichael et al., 2015 for details). Participants were provided with the URL of our online interface and completed the test in their own time.

Our replication of the *Synesthesia Battery* first obtains consent for testing and then records demographic information about participants including age and sex. Participants are then asked whether they experience grapheme-colour synesthesia with the question “Do numbers or letters cause you to have a colour experience?” A checkbox is provided for participants to record separately whether these colours are triggered automatically by numbers and/or digits. If participants indicated that they saw neither letters nor numbers in colour, they advanced to an exit page thanking them for their participation.

The consistency test was completed by participants who answered in the affirmative to having coloured letters/digits. This test displays individually on-screen the letters A-Z and/or the digits 0–9 (according to how participants responded to the checkboxes described above). Each grapheme is shown three times in a random order, and on each display, participants must indicate their synaesthetic colour by selecting it from an on-screen palette of 256x256x256 colours. The program compares the colour selected each time the same grapheme was presented (e.g., it compares the three colours for the letter A). It then produces a standardised score to reflect how far away in colour space those three colours were, averaged across all graphemes. A small standardised score reflects consistent colours (i.e., selections for the same grapheme were close in colour-space). A score less than 1 indicates the high level of consistency typical of a synaesthete and this is the diagnostic threshold in this test. For full details regarding how this test is designed and implemented, please refer to Eagleman et al. (2007).

### Results

In our study, we classified as non-synaesthetes all those who were directed to the early-exit page (i.e., those who said they did not experience coloured letters and/or digits) and all those who continued but scored 1 or higher. The remainder were classified as synaesthetes (i.e., those who scored <1).

From 3893 participants, we identified 54 grapheme-colour synaesthetes with coloured letters and/or digits (n = 5 with coloured letters; n = 26 with coloured digits; n = 23 with both coloured letters and digits), giving an overall prevalence of 1.39%. Of these 54 synaesthetes, 33 were female and 21 were male.^3^^3^Of the 33 female synaesthetes, 12 reported both coloured letters and digits, 5 reported experiencing coloured letters only and 16 reported coloured digits only. Of the 21 male, 11 reported both coloured letters and digits, 0 reported coloured letters only and 10 reported coloured digits only. Calculating the overall prevalence of grapheme-colour synaesthesia for each sex separately taking into account the total number of men and women tested (2135 and 1758 respectively) gives us a female prevalence of 1.55% and a male prevalence of 1.19%, producing a female: male ratio of 1.3:1. This difference in the ratio of female versus male synaesthetes is not significant (χ^2^ = 0.63, *df* = 1, *p* = 0.43).^4^^4^If we examine the sex ratio for only synaesthetes who have both coloured letters AND numbers (n = 23; 12 female) we find a prevalence of 0.59%. Calculating the synaesthesia prevalence of each sex separately gives us a female prevalence of 0.56% and a male prevalence of 0.63%, and a ratio of 0.89: 1. Using a chi-squared test, we again determined the difference in ratio of female versus male synaesthetes is not significant (χ2 = 0.002, df = 1, p = 0.962).

### Bayesian analysis

To further investigate our null result, we performed two types of Bayesian analyses below. Together these suggest that our sufficiently powered investigation of whether there is a 6:1 ratio gave strong support for the null hypothesis. However, they also provide an estimate of how small any possible female bias might yet be.

First, our Bayes factors analysis allows us to evaluate to what extent the data supports the hypothesis under investigation against the null hypothesis (Rouder, Speckman, Sun, Morey & Iverson, 2009). Following Jeffreys (1961), a Bayes factor of less than 0.33 provides strong support for the null hypothesis, a Bayes factor >3 provides support for the alternative hypothesis and values in between indicate no firm conclusions should be drawn. Our Bayes factor was 0.014, indicating strong support for the null hypothesis that sex does not significantly influence the prevalence of grapheme-colour synaesthesia.

Exploring our data further, a second analysis suggests that although there was no large significant difference across the sexes, there may yet be small difference, and we can calculate its size. We constructed a beta-binomial model of our acquired data which shows that any difference between the numbers of male and female synaesthetes in the general population is likely to be very small. Calculating a 95% confidence interval of the difference in prevalence, we see any difference in prevalence between females and males is likely to fall in the range −0.4% to 1.1%. Theoretically speaking, therefore, if we were confident that—say—our male prevalence of 1.19% were correct, we would therefore be 95% sure that the true female prevalence is in the small range between 0.79% (1.19%–0.4%) to 2.29% (1.19%+1.1%). Indeed, if there were a difference between men and women, our beta-binomial model also shows there is an 82% chance that the prevalence would be higher for females—albeit to this very marginal degree.

### Discussion

We investigated the prevalence of grapheme-colour synaesthesia in males and females to challenge the suggestion that there are six times more female synaesthetes than male in the general population (Barnett et al., 2008; Baron-Cohen et al., 1996; Rich et al., 2005). First we pointed out that two previous studies showing this level of strong bias reported that their methodology relied on self-referral (e.g., Baron-Cohen et al., 1996; Rich et al., 2005). This method likely encouraged female synaesthetes to reply more than males (Simner et al., 2006; following Dindia & Allen, 1992). Second, we described how previous studies *not* liable to this confound (e.g., Simner et al., 2009, 2006) had *not* found a strong 6:1 bias towards females, and indeed had found no significant difference across the sexes at all. Third we examined an additional study showing a 6:1 bias of females which claimed not to rely on self-referral (Barnett et al., 2008). Using their published data and descriptions of study, we suggested that they may not have taken into account the total number of males/females tested overall or may not have used objective tests to verify synaesthesia in all participants, and that their methods did not appear to be entirely free of the self-referral confound.

In our empirical investigation, we screened 3893 individuals for grapheme-colour synaesthesia following a power analysis. We took care to avoid self-referral confounds and we individually tested every member of a pre-determined cohort with an objective test for synaesthesia. We found that 33 out of the 2135 females tested had grapheme-colour synaesthesia (for coloured letters and/or digits; female prevalence 1.55%) as well as 21 out of 1758 males (male prevalence 1.19%). This ratio of 1.3: 1, female to male synaesthetes, was non-significant. Further Bayes analyses suggest support for the null result in our data, but that there remains the possibility of a very small sex differences, in the range of −0.4% to 1.1%, with a female bias being more likely than a male bias.

Our results largely corroborate the findings of our previous comparison study, Simner et al. (2006) which reported an overall prevalence of grapheme-colour synaesthesia of 2%, compared to 1.39% in our own study (and this difference is non-significant; χ2 = 1.16, p = .28). This was for synaesthetes with coloured letters and/or digits, but it is also possible to directly compare our findings in the female: male ratio if we consider synaesthetes with both coloured letters *and* digits (since this is the type of sex data reported in Simner et al., 2006). In this comparison we find a female: male ratio of 0.9:1 in the current study compared to an identical ratio (0.9:1) found in Simner et al. (2006; their female prevalence was 1.03% and their male prevalence was 1.15%).

In our calculations we point out that we classified participants as synaesthetes according to the conventional cut-off, as stated within the test we used by Eagleman et al. (2007). This conventional cut-off for synaesthesia is a score <1. Two recent studies however have suggested that a more accurate approach might be a cut-off centred on 1.43 rather than 1 (for details see Carmichael et al., 2015; Rothen, Seth, Witzel & Ward, 2013). For this reason, we also re-calculate our prevalence and female/male ratio according to the 1.43 cut-off and find a yet-closer female: male ratio in synaesthesia. For clarity to aid the reader, we have presented this data along with our other prevalence/ratios in Table 1.

**TABLE 1:** Shows the number of confirmed male (M) and female (F) grapheme-color synaesthetes found in our total sample of 3893 subjects (F = 2135; M = 1758). The prevalences are shown in brackets, with the female: male ratio beneath. This is done twice according to two different cut-off for synaesthesia (a score of 1 vs. 1.43 in *The Synesthesia Battery*) and twice according to two different definitions of grapheme-color synaesthesia (having colored letters AND/OR digits, vs. colored letters AND digits).

Coloured triggers	Sex & ratio	Battery cut-off at 1	Battery cut-off at 1.43
Letters AND/OR digits	F	33 (1.55%)	55 (2.58%)
	M	21 (1.19%)	39 (2.22%
	F + M	=54 (1.39%)	=94 (2.42%)
	**Ratio F:M**	**1.3:1**	**1.2:1**
Letters AND digits	F	12 (0.56%)	23 (1.08%)
	M	11 (0.63%)	19 (1.08%)
	F + M	=23 (0.59%)	=42 (1.08%)
	**Ratio F:M**	**0.89: 1**	**1:1**

Of course we point out that our findings relate only to the sex ratio and prevalence of the population we sampled, and the type of synaesthesia we investigated. We note that our average sampled participant was 28 years old, which is younger than the national average (median = 40.5 years; Central Intelligence Agency, 2014), and this might have influenced the prevalence we generated. Furthermore, we looked only at grapheme-colour synaesthesia, which is just one of many variants of the condition (see Cytowic & Eagleman, 2009; Day, 2005). A recent study of a very large number of self-referred synaesthetes by Novich, Cheng and Eagleman (2011) revealed that groups of variants clusters into synaesthetic subtypes (e.g., people with grapheme-colour synaesthesia are likely to have a second form involving colour, but not taste). This suggests there may be multiple forms of the condition, and indicates in turn that what is true of grapheme-colour synaesthesia (e.g., its sex ratio) may not be representative of all synaesthesias.

One curiosity not yet understood is the apparent extent of the female bias in self-referral studies for synaesthesia. We have shown there are roughly equivalent numbers of female to male grapheme-colour synaesthesia in the general population—or at the very most, that there are only 1.3 women for every man. However, six times more female synaesthetes are detected in self-referral studies (e.g., Baron-Cohen et al. 1996). We attribute this difference in part to the known confound that promotes responses from women over men in self-referral (e.g., Dindia & Allen, 1992; Rosenthal & Rosnow, 1975). However, Barnett et al. (2008) point out that this bias usually gives just a slight variation of around 10% (Dindia & Allen, 1992). Why then might the female bias be so exaggerated in studies of synaesthesia—and indeed, why are the rates so consistent across self-referral studies? It could be, for example, that female synaesthetes—although not greatly more common—have perhaps more intense experiences or are more aware of their synaesthesia or attend to it more in daily life. This might make them more likely to self-refer. However, we have no data to support any specific supposition in this area, so leave this question for future investigations.

Understanding sex ratios are important in understanding the origins of synaesthesia. Initial findings that synaesthesia appeared more common in females led to suggestions that synaesthesia was either not fully expressed in males or that it was linked to the X-chromosome in some way (Bailey & Johnson, 1997; Baron-Cohen et al., 1996; Ward & Simner, 2005). Indeed, the extent of the female bias led researchers to propose that synaesthesia might causes lethality in males *in utero* (Baron-Cohen et al., 1996). Subsequent research, including our own study here, suggests this is not the case. In combination with previous studies from our own lab and elsewhere, we conclude there no very strong female bias (Simner et al., 2009, 2006), that families containing synaesthetes are equally likely to produce female or male offspring (Barnett et al., 2008; Ward & Simner, 2005), that there are confirmed cases of male-to-male transmission (Asher et al., 2009), and one case of monozygotic male twins who are discordant for synaesthesia (Smilek, Dixon & Merikle, 2005). Finally, neither Asher et al. (2009) nor Tomson et al. (2011) found evidence for a major locus on the X chromosome in their genome-wide studies.^5^^5^Both these genetics studies screened probands and family members for synaesthesia but no information about the sex of participants was given in Asher et al. (2009). Although Tomson et al. (2013) did report the sex of their participants, they screened just 5 families giving too few datapoints to draw firm conclusions from their female: male ratio (2.7:1) which was, furthermore, generated via a self-referral bias in probands. These genetics studies therefore serve an important purpose in describing the genetic aetiology of synaesthesia but cannot be used to empirically authenticate sex-ratios. This suggests a need to revisit our early understanding of the mode of inheritance of synesthesia (see Asher & Carmichael, 2013, for review) and we provide our data for future studies to do so.

We finally point out that our own studies have shown relatively flat distributions of synaesthesia in men and women, with a slight male bias when considering grapheme-colour synaesthetes with coloured letters *and* digits (female: male ratio of 0.9: 1 both here and in Simner et al., 2006) and a slight female bias when considering grapheme-colour synaesthetes with coloured letters *and/or* digits (here, female: male ratio of 1.3: 1). It may yet be possible to estimate the numbers required to test this much reduced difference across the sexes (e.g., power analyses in the ratio of 1.3:1 suggest we would need to screen 47516 participants) but for the current study we have shown that there is no 6:1 ratio of female to male synaesthetes, even with sufficient power to test for such a difference.
